# Cancer-Associated Fibroblasts Hinder Lung Squamous Cell Carcinoma Oxidative Stress-Induced Apoptosis via METTL3 Mediated m^6^A Methylation of COL10A1

**DOI:** 10.1155/2022/4320809

**Published:** 2022-10-06

**Authors:** Yuchan Li, Xiaoxue Li, Muwen Deng, Changda Ye, Yuanhong Peng, Yan Lu

**Affiliations:** ^1^Department of 2nd Oncology, Guangdong Second Provincial General Hospital, Guangzhou, 510317 Guangdong, China; ^2^Institute of Pathology, University Medical Center, Göttingen, Germany; ^3^Department of Gastrointestinal Surgery, Shunde Hospital, Southern Medical University (The First People's Hospital of Shunde Foshan), Foshan, 528300 Guangdong, China; ^4^The Second School of Clinical Medicine, Southern Medical University, Guangzhou, 510080 Guangdong, China; ^5^GCP Center, Shunde Hospital, Southern Medical University (The First People's Hospital of Shunde Foshan), Foshan, 528300 Guangdong, China

## Abstract

**Background:**

Cancer-associated fibroblasts (CAFs) within the tumor microenvironment are key players in tumorigenesis and tumor development. Nevertheless, the regulatory mechanisms of CAFs on lung squamous cell carcinoma- (LUSC-) associated remain poorly elucidated.

**Methods:**

The microarray dataset GSE22874, containing 30 specimens of primary culture of normal fibroblasts (NFs) and 8 specimens of cancer-associated fibroblasts (CAFs) samples derived from LUSC, was retrieved from the Gene Expression Omnibus (GEO) database and then calculated by using the R language (limma package) to identify differentially expressed genes (DEGs). CAF-conditioned medium (CAF-CM) was collected and used to culture LUSC cells, followed by assessment of cell proliferation, apoptosis, and oxidative stress levels by using CCK-8, annexin V-FITC/PI double staining and ELISA assays. Subsequently, COL10A1 was knocked down in CAFs to assess the role of COL10A1 in CAF regulation of LUSC behavior. Bioinformatics online analysis and MeRIP were applied to predict and test the m^6^A modification of COL10A1 mRNA and the regulatory relationship with METTL3. Rescue experiments were next performed to explore the effects of METTL3 and COL10A1 in CAFs on LUSC cell proliferation, apoptosis, and oxidative stress. LUSC tumor cells with or without (COL10A1-silenced) CAFs were subcutaneously inoculated in nude mice to evaluate the effect of COL10A1 in CAFs on LUSC tumor growth.

**Results:**

Elevated expression of COL10A1 was found in LUSC-derived CAFs by GSE22874 dataset analysis. We discovered that COL10A1 and METTL3 was expressed in both LUSC cells and matched CAFs, while COL10A1 expression was prominently higher in CAFs than in LUSC cells. CAF-CM memorably encouraged LUSC cell proliferation and suppressed apoptosis-induced oxidative stress, which was reversed by interfering with COL10A1 expression in CAFs, suggesting that COL10A1 might be secreted by CAFs into the culture medium to exert its effects inside LUSC cells. Global m^6^A modification was decreased in METTL3 knocked down CAFs. M^6^A modification, expression levels, and stability of COL10A1 mRNA were impaired upon METTL3 knockdown in CAFs. Overexpression of COL10A1 in CAFs partially reversed the effect of METTL3 knockdown on the malignant behavior of LUSC cells. *In vivo* studies confirmed that CAFs accelerated LUSC tumor growth, and this effect was counteracted by COL10A1 silencing.

**Conclusions:**

COL10A1 secreted by CAFs could facilitate LUSC cell proliferation and repress apoptosis-induced oxidative stress, and the mechanism was due to elevated expression mediated by METTL3 promoting its mRNA m^6^A modification, thereby accelerating tumor growth.

## 1. Introduction

Lung cancer is one of the most common malignant diseases with high morbidity and mortality, and the incidence is increasing with each passing year [1]. Depending on the pathological type, lung cancer can be divided into non-small-cell lung cancer (NSCLC) and small cell lung cancer (SCLC), of which 85% of lung cancer patients belong to NSCLC [2]. NSCLC in turn includes two main pathological types, lung adenocarcinoma (LUAD) and lung squamous cell carcinoma (LUSC). LUSC, as one of the major pathological types of non-small-cell lung cancer, leads to the death of approximately 400000 patients annually worldwide [3]. Despite the tremendous advances in existing medical treatments for the diagnosis, treatment, and care of LUSC, the mortality rate of LUSC has risen rapidly over the past few decades, and the overall survival rate is still very low. The reason for the poor survival rate and poor prognosis is that a large proportion of LUSC patients are already in the middle to advanced stages of lung cancer at the time of diagnosis and may already present with cancer metastasis [4]. It follows that the key to improving the survival rate of LUSC patients is the screening and detection of early stage.

Tumors are complex structures composed of malignant cells and a large number of non-neoplastic cells that interact with each other to create a tumor microenvironment (TME) [5]. The TME is mainly composed of blood vessels, extracellular matrix (ECM), and other non-neoplastic cells (including fibroblasts, adipocytes, and vascular endothelial cells, as well as immune cells such as T lymphocytes, B lymphocytes, NK cells, and tumor associated macrophages) surrounding the tumor, as well as cytokines and exosomes secreted by the cells [6]. Among them, cancer-associated fibroblasts (CAFs) are an important class of cellular components in TME, which have a wide range of origins and can be transformed by resident fibroblasts, BMSCs and HSCs, adipose stem cells, endothelial cells, and stellate cells in hepatocytes [7]. In addition to producing growth factors and inflammatory factors to modulate fibroblast activation in an autocrine manner, CAFs have been found to regulate the behavior of tumor cells and other stromal cells in a paracrine manner, to recruit them to the primary lesion or metastatic lesions of cancer, and to remodel the ECM and build the TME, ultimately promoting tumor cell proliferation, invasion, metastasis, drug resistance, and so on [8]. A study found that CAF-derived exosomes could suppress peripheral blood mononuclear cell-induced lung cancer cell killing and promote lung cancer progression [9]. Further, HIF-1*α* in CAFs could activate the NF-*κ*B signaling pathway and enhance the subsequent secretion of CCL5, thereby promoting lung cancer tumor growth [10]. A data displayed that conditioned medium (CM) of CAFs could raise lung cancer cell proliferation, migration, and invasion, whereas blocking VCAM-1 in CAF-CM attenuated cancer cell proliferation and invasion [11].

Oxidative stress is an imbalance between free radicals and reactive metabolites that produces a large number of oxidative intermediates, leading to damage of important biomolecules and cells with potential implications for the whole organism [12]. Since oxidative stress can increase DNA mutations or induce DNA damage leading to genomic instability and cell proliferation, cancer initiation and progression are associated with oxidative stress [13]. From the whole apoptotic process, initial stress-induced cell damage cannot kill cells directly, but is able to trigger apoptotic signaling that leads to cell death [14]. Liu et al. found that oxidative stress inhibited the growth and induced apoptosis of human U251 glioma cells through a caspase-3-dependent pathway [15]. Tor et al. demonstrated that ethyl acetate extract induced apoptosis of breast cancer cells and its chemical features through oxidative stress generating, mitochondria dependent, and caspase independent pathways [16]. ROS appear to be important regulatory signals, as proposed by the growth suppressive role of SOD [17]. In fibroblasts, SOD inhibitors seem to favor apoptosis over cell proliferation. Recombinant adenoviral vectors expressing the radical scavenging enzymes Mn SOD and Cu, Zn SOD were able to reduce the level of apoptosis [18]. SOD has been reported to provide protection against tumor necrosis factor (TNF) cytotoxicity in hematopoietic cells [19]. TNF-induced antiproliferative effects, caspase-3 activation as well as other indicators of apoptosis were also completely inhibited by SOD activity [20]. Hypoxia-induced CAFs convey cisplatin resistance to sensitive NSCLC cells by delivering PKM2, which in turn inhibits oxidative stress-induced apoptosis [21]. Nevertheless, whether oxidative stress is involved in CAF involvement in LUSC progression remains to be investigated.

The aim of this study was to investigate the effects and molecular mechanisms of LUSC-associated CAFs on LUSC (SW900 and LOU-NH91 cells) in terms of cell proliferation, oxidative stress, and apoptosis and to identify the key genes in CAFs combined with bioinformatics software and biological experiment validation.

## 2. Materials and Methods

### 2.1. Tissue Specimens

This study enrolled 43 LUSC patients in The First People's Hospital of Shunde Foshan from May 2019 to August 2021. None of these patients received preoperative chemotherapy or radiotherapy. Subsequently, isolation and culture of CAFs and NFs were performed in cancer tissues and corresponding noncancerous tissues. Informed consent was obtained from patients accordingly to established protocols approved by the institutional review board of The First People's Hospital of Shunde Foshan. This study conformed to the declaration of Helsinki.

### 2.2. Ethics Approval

Both human and animal studies were conducted after approval from the committees of clinical ethics of The First People's Hospital of Shunde Foshan.

### 2.3. Cell Culture

LUSC cell lines SW900 and LOU-NH91 (ATCC, Manassas, VA, USA) were cultured at 37°C in a 5% CO_2_ saturated humidified cell culture incubator with 10% fetal bovine serum (FBS, Gibco, CA). CAFs were cultured until 70-90% confluent, at which time the medium used was collected and passed through a 0.22 *μ*M filter and diluted 1 : 1 with RPMI-1640 containing 10% FBS. RPMI1640 medium supplemented with 10% FBS as the control medium. LUSC cells were cultured in conditioned media from CAFs cells for 21 days.

### 2.4. Bioinformatics Analysis

Microarray data are available with the accession numbers GSE22874 from Gene Expression Omnibus database (GEO, https://www.ncbi.nlm.nih.gov/geo/). The datasets GSE22874 was based on the platform GPL5175, including 30 normal fibroblasts (NFs) primary culture samples and 8 carcinoma-associated fibroblasts (CAFs) primary culture samples derived from LUSC. The difference analysis was performed using the limma package based on the R software, which employs the classical Bayesian's *t*-test analysis method with filtering criteria: |log fold change| ≥ 0.5, adjust *P* < 0.05. Targeted visualization of differential sites. The volcano plot was drawn by using the R language ggplot2 package to demonstrate the differentially expressed genes. Then, the Heatmap was plotted against the significantly upregulated versus significantly downregulated differentially expressed genes using the pHeatmap package in the R language, and samples were clustered on the upper part of the Heatmap, and sites were clustered on the left side of the Heatmap. In addition, m^6^A modification sites of COL10A1 were predicted by online analysis website (http://www.cuilab.cn/sramp).

### 2.5. RT-qPCR Assay

Each group of differently treated cells were collected, and then TRIzol (Thermo Fisher, USA) was added to extract total cellular RNA. Total cellular RNA was reverse transcribed into cDNA by using a reverse transcription kit. RT-qPCR was performed by using cDNA as template, and then PCR products were detected with real time PCR system (Thermo Fisher, USA) with three replicate wells set for each group. The primer sequences were as follows: COL10A1 forward, 5′-TAT CCC GGC CCT ACT CCA AA-3′; and reverse, 5′-TTC AGC ACA GAG TCA GGC AG-3′. The relative expression levels were calculated by using 2^−ΔΔCT^ method. We used GAPDH as endogenous control.

### 2.6. Western Blotting

Total cell protein was extracted with RIPA lysate, and the protein concentration was determined using a BCA protein assay kit (Thermo Fisher, USA) in a microplate reader. After denaturation for 10 min with the addition of loading buffer, 50 *μ*g of protein samples were subjected to SDS-PAGE and transferred onto PVDF membranes. The membrane was blocked with blocking solution (5% nonfat dry milk) for 2 h and subsequently washed three times by using TBST buffer. Then, the membranes were incubated with horseradish peroxidase- (HRP-) conjugated goat antirabbit IgG (1 : 3000, Abcam, ab6721) for 1 h at room temperature. The secondary antibody was added by using TBST washing three times. This was followed by incubation on a shaker and protein exposure. ImageJ software was applied to detect and analyze the gray values of protein bands on the membrane. *β*-Actin was used as an endogenous control.

### 2.7. Cell Apoptosis Assay

Cell apoptosis was detected by using flow cytometry. The cells to be tested were washed 2 times with phosphate buffered saline, then 5 *μ*l of annexin V dye solution and 10 *μ*l of propidium iodide dye solution were added to stain for 30 min in the dark at 4°C. The apoptosis rate was detected by using flow cytometry.

### 2.8. Genetic Overexpression and Knockdown

The COL10A1 full sequence was ligated into pcDNA3.1 plasmid (GenePharma, China). The shRNAs were designed by QIAGEN to knock down METTL3 and COL10A1. Confluent cells were diluted in DMEM medium, and the cells were observed to grow to about 70% confluence when the cell monolayer was covered with serum-free DMEM medium. The plasmid transfection was performed using TurboFect Transfection Reagent as required for the experiments. All cells in each group were collected for subsequent experiments after incubating in an incubator at 37°C with 5% CO_2_ for a specified period of times.

### 2.9. CCK-8 Assay

Cells were seeded in 96 well culture plates at a cell number per well of 3 × 10^3^, and 5 replicate wells were set in each group. Transfection was performed after incubation at 37°C in 5% CO_2_ until the cells became adherent. Then, the cells were incubated at 37°C in a 5% CO_2_ incubator for 0, 24, 48, and 72 h after which the supernatant was discarded and 100 *μ*l of complete medium and 10 *μ*l of CCK-8 (Sigma-Aldrich, St. Louis, MO, USA). After incubation at 37° C in 5% CO_2_ for 1 h, the optical density (OD) value at 450 nm was measured using a microplate reader (Bio-Rad, Hercules, CA, USA).

### 2.10. RNA Stability Assay

Actinomycin D (act D, 5 *μ*g/ml) was added to the cells. Total RNA was isolated and RT-PCR was performed to measure relative levels of COL10A1 as previously described.

### 2.11. Total m^6^A Measurement

Total RNA from cells in each group was isolated using TRIzol (Thermo Fisher, USA) according to the manufacturer's instructions. M^6^A content was subsequently quantified using the EpiQuik m^6^A RNA Methylation Quantification Kit (EpiGentek, USA).

### 2.12. M^6^A RNA Immunoprecipitation Assay (MeRIP)

Total RNA from cells of each group was isolated, and an mRNA purification kit (Thermo Fisher, USA) was used to further enrich poly (A)+RNA, which was then treated using DNase I (Thermo Fisher, USA). Subsequently, the Magna methylated RNA immunoprecipitation (MeRIP) m^6^A kit (Thermo Fisher, USA) is employed to incubate global RNA with m^6^A antibodies for immunoprecipitation. Finally, RT-PCR was performed on extracted RNA using mRNA primers of COL10A1 and then normalized to input.

### 2.13. Enzyme-Linked Immunosorbent Assay (ELISA)

Cells were assayed for ROS, SOD, and GPX contents with ELISA kits (Thermo Fisher Scientific, Waltham, Ma, USA) according to the manufacturer's instructions.

### 2.14. In Vivo Experiments

5-week-old male athymic BALB/C nude mice were obtained from experimental animal center of Southern Medical University, and subsequently randomly divided into four groups, including SW900, SW900+CAFs, and SW900+CAFs^COL10A1shRNA^. First, we stably transfected CAFs with COL10A1 shRNA. For xenograft experiments, SW900 or/and CAFs (5 × 10^5^ cells/mouse) were subcutaneously injected into the right armpits of mice. Tumor length and width were calculated with vernier calipers every 7 days. After 35 days, the mice were humanely sacrificed, and the subcutaneous tumors were excised and removed. Experimental protocols were performed in accordance with the National Institute of Health guidelines for the Care and Use of Laboratory Animals.

### 2.15. Statistical Analysis

SPSS 22.0 and GraphPad prism 7.0 were used for data analysis and mapping. The pairwise comparisons were analyzed using the chi-square test. The measurement data were represented as mean ± SEM with normal distribution and homogeneity of variance. Student's *t*-test was used for comparison of two samples. The means of the different groups were compared using one-way or two-way analysis of variance (ANOVA) following Tukey's post hoc test. *P* < 0.05 was considered as statistically significant difference. All experiments were repeated 3 times (*N* = 3).

## 3. Results

### 3.1. Elevated Expression of COL10A1 in CAFs of LUSC Based on GEO Dataset Mining

**Figure 1:**
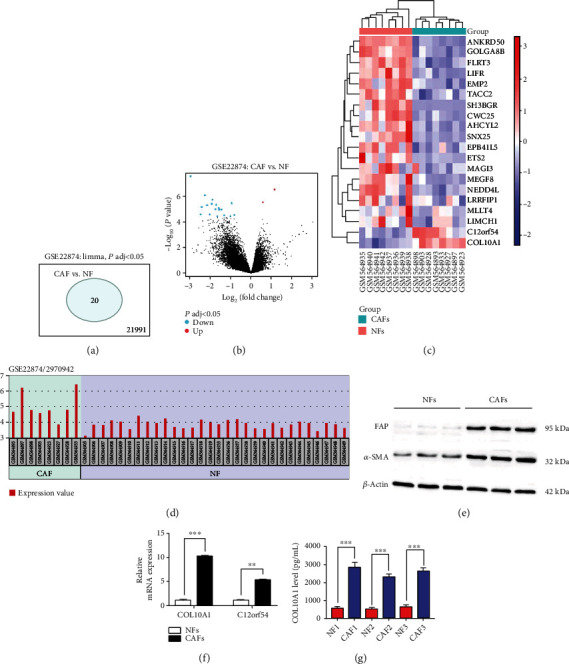
Elevated expression of COL10A1 in CAFs of LUSC based on GEO dataset mining. (a and b) Volcano plot of differentially expressed genes (DEGs) in GSE22874 dataset, which included 30 normal fibroblasts (NFs) primary culture samples and 8 carcinoma-associated fibroblasts (CAFs) primary culture samples derived from LUSC. (c) Heatmap visualization analysis of 20 DEGs. (d) Expression of COL10A1 in individual samples of the GSE22874 dataset. (e) Western blotting was employed to examine the expression of FAP and *α*-SMA in CAFs and NFs derived from three different LUSC samples. (f) RT-qPCR was used to test the expression of COL10A1 and C12orf54 in CAFs and NFs. (g) ELISA was employed to examine the expression of COL10A1 in CAFs and NFs derived from three different LUSC samples. Data were presented as mean ± SEM. *N* = 3. ^∗∗^*P* < 0.01, ^∗∗∗^*P* < 0.001.

Analysis of dataset GSE22874 downloaded from the Gene Expression Omnibus (GEO) database, including 30 normal fibroblasts (NFs) primary culture samples and 8 carcinoma-associated fibroblasts (CAFs) primary culture samples derived from LUSC. In addition, GSE22874 dataset was found to have a total of 20 differentially expressed genes (DEGs) by screening (Figure 1(a)). The gene expression profile was next analyzed in the GSE22874 dataset, generating a volcano plot and Heatmap of DEGs, which revealed 2 significantly upregulated DEGs and 18 significantly downregulated DEGs in CAFs (Figures 1(b) and 1(c)). We found that COL10A1 was prominently highly expressed in CAFs (Figure 1(d)). Subsequently, to further confirm the predicted results, we isolated and primary cultured LUSC-derived CAFs and matched NFs *in vitro.* Validation of CAF surface markers revealed that the expression of fibroblast-activating protein (FAP) and alpha-smooth muscle actin (*α*-SMA) was substantially elevated in CAFs compared with NFs (Figure 1(e)). In addition, we also discovered that both COL10A1 and C12orf54 were significantly upregulated in CAFs, with COL10A1 being more differentially expressed (Figures 1(f) and 1(g)).

### 3.2. CAF-CM Prominently Encouraged LUSC Cell Proliferation and Suppressed Apoptosis-Induced Oxidative Stress

**Figure 2:**
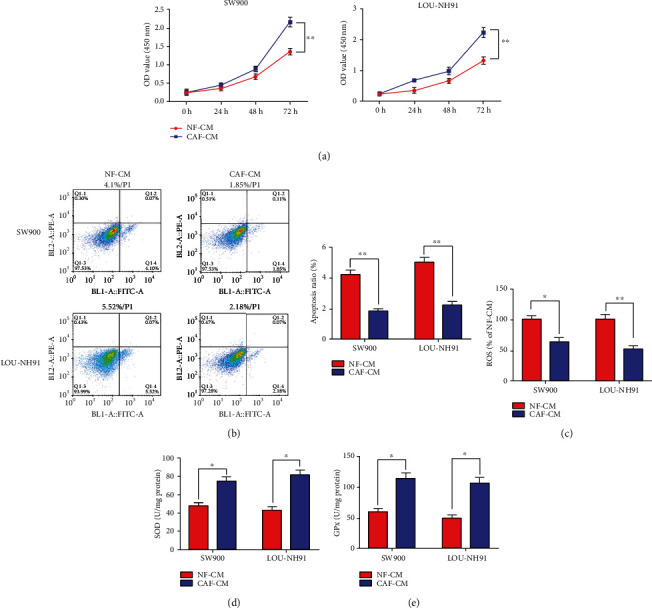
CAF-CM prominently encouraged LUSC cell proliferation and suppressed apoptosis-induced oxidative stress. (a) The CCK-8 kit was used to assess cell proliferation after CAF-CM treatment of SW900 and LOU-NH91 cells for 72 h. (b) The annexin V-FITC/PI double staining assay was used to test cell apoptosis after CAF-CM treatment of SW900 and LOU-NH91 cells for 48 h. (c–e) ELISA was employed to examine contents of ROS, SOD, and GPX after CAF-CM treatment of SW900 and LOU-NH91 cells for 48 h. Data were presented as mean ± SEM. *N* = 3. ^∗^*P* < 0.05, ^∗∗^*P* < 0.01.

Next, we employed the conditioned medium of CAFs (CAF-CM) to culture different cell lines of l LUSC to explore whether CAFs had an effect on the malignant behavior of LUSC cells SW900 and LOU-NH91. The growth curve generated by the CCK-8 assay displayed a significant elevation of the cell OD value after culturing the SW900 and LOU-NH91 cells by using CAF-CM, indicating cell proliferation was increased (Figure 2(a)). Further, apoptosis results revealed that the apoptotic rate of SW900 and LOU-NH91 cells were prominently decreased after CAF-CM treatment (Figure 2(b)). Subsequently, we detected ROS, SOD, and GPX contents in cell supernatants using ELISA to evaluate the regulation of CAFs on oxidative stress levels in LUSC cells. The results revealed that CAF-CM treatment significantly inhibited ROS content and facilitated SOD and GPX generation (Figures 2(c)–2(e)).

### 3.3. Knockdown of COL10A1 in CAFs Impedes the Promotion of LUSC Cell Growth by CAF-CM and Restraint of Apoptosis-Induced Oxidative Stress

**Figure 3:**
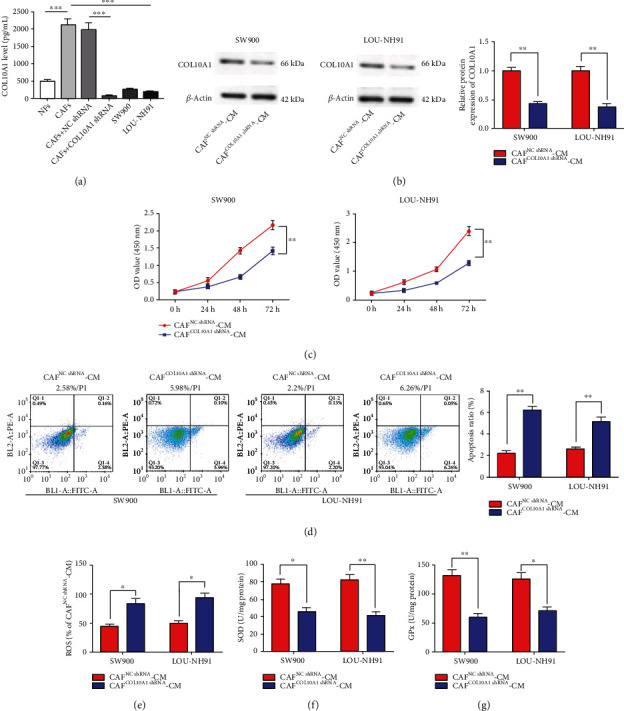
Knockdown of COL10A1 in CAFs impedes the promotion of LUSC cell growth by CAF-CM and restraint of apoptosis-induced oxidative stress. (a) ELISA was employed to examine COL10A1 level in NFs, CAFs, CAFs transfected with NC shRNA or COL10A1 shRNA, SW900 cells, and LOU-NH91 cells. (b) Western blotting was applied to test COL10A1 protein level in SW900 and LOU-NH91 cells treated with CAF^NCshRNA^-CM and CAF^COL10A1shRNA^-CM. (c) The CCK-8 kit was used to assess cell proliferation, which treated with CAF^NCshRNA^-CM and CAF^COL10A1shRNA^-CM. (d) The annexin V-FITC/PI double staining assay was used to test cell apoptosis in SW900 and LOU-NH91 cells treated with CAF^NCshRNA^-CM and CAF^COL10A1shRNA^-CM. (e–g) ELISA was employed to examine contents of ROS, SOD and GPX in SW900 and LOU-NH91 cells treated with CAF^NCshRNA^-CM and CAF^COL10A1shRNA^-CM. Data were presented as mean ± SEM. *N* = 3. ^∗^*P* < 0.05, ^∗∗^*P* < 0.01, ^∗∗∗^*P* < 0.001.

Subsequently, to further confirm whether the CAFs effects on LUSC cells were through the involvement of COL10A1, COL10A1 shRNA was transfected into CAFs, and then CAF^COL10A1shRNA^-CM were collected and treated with SW900 and LOU-NH91 cells. ELISA results showed that COL10A1 expression was significantly reduced within CAFs after COL10A1 shRNA transfection (Figure 3(a)). In addition, COL10A1 expression was apparently lower in SW900 and LOU-NH91 cells than in CAFs (Figure 3(a)). Further, compared with CAF^NCshRNA^-CM, CAF^COL10A1shRNA^-CM was able to reduce the expression of COL10A1 in SW900 and LOU-NH91 cells (Figure 3(b)), suggesting that COL10A1 may be secreted by CAFs into the culture medium and then into SW900 and LOU-NH91 cells. Next, we further discovered that CAF^COL10A1shRNA^-CM treatment dramatically repressed cell proliferation, and contents of SOD and GPX in the cell supernatant, as well as accelerated cell apoptosis and ROS levels (Figures 3(c)–3(g)).

### 3.4. METTL3 Stabilizes COL10A1 Expression by Elevating its m^6^A Modification in CAFs

**Figure 4:**
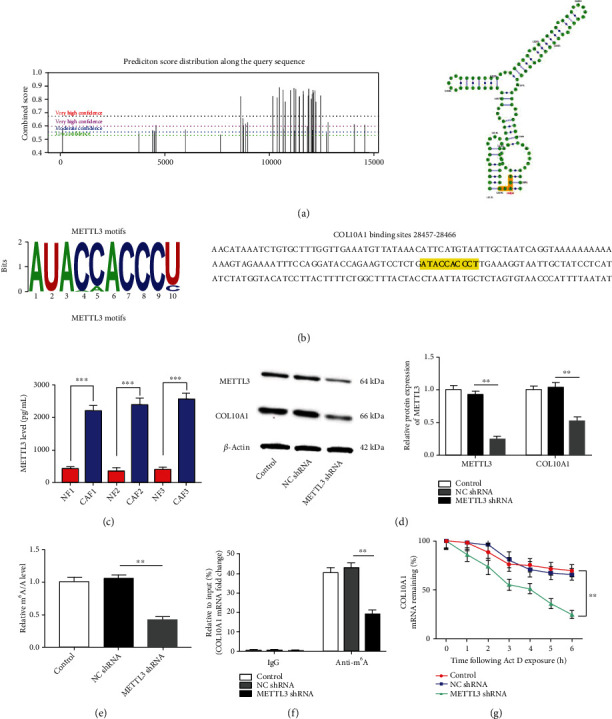
METTL3 stabilizes COL10A1 expression by elevating its m^6^A modification in CAFs. (a) Diagram of m^6^A modification peaks on COL10A1 mRNA, and schematic of the most likely binding sites. (b) Motifs of METTL3 and the binding sequences of its on COL10A1 3′UTR. (c) Protein content of METTL3 in NFs and CAFs was detected by ELISA. (d) The protein levels of METTL3 and COL10A1 in CAFs after transfection with METTL3 shRNA were tested by Western blotting. (e) MeRIP was used to analyze m^6^A modification levels on COL10A1 in CAFs. (f) m^6^A quantitative analysis was applied to analyze m^6^A global levels in CAFs. (g) Actinomycin D (5 *μ*g/ml) was added to the cells to assess mRNA stability of COL10A1. Data were presented as mean ± SEM. *N* = 3. ^∗∗^*P* < 0.01, ^∗∗∗^*P* < 0.001.

Next, we aimed at exploring the underlying mechanism by which COL10A1 is highly expressed in CAFs. Prediction of whether COL10A1 is modified via m^6^A on mRNA by online bioinformatics tools (http://www.cuilab.cn/sramp).  Figure 4(a) displayed that there were m^6^A binding sites ′GGACT′ on mRNA of COL10A1, among them, the highest confidence binding sites were at 12151-12155 on COL10A1. To further demonstrate whether m^6^A methylation on COL10A1 is regulated by RNA methyltransferases, we obtained the binding motifs of METTL3, METTL14, and WTAP at Starbase website (https://starbase.sysu.edu.cn/) and retrieved the mRNA 3′UTR sequences of COL10A1. The results displayed that the possible binding sites of METTL3 and COL10A1 were ATACCACCCT (Figure 4(b)). Because no binding sites were found for COL10A1 with METTL4 and WTAP, they are not shown in figures. Interestingly, we found that METTL3 level was elevated in LUSC derived CAFs compared with NFs (Figure 4(c)). Subsequently, METTL3 was knocked down in CAFs to assess its effect on COL10A1 expression and mRNA stability.  Figure 4(d), shown as the interference efficiency of METTL3 shRNA, indicates that METTL3 shRNA can suppress the METTL3 expression. MeRIP results confirmed that m^6^A antibody enrichment on COL10A1 mRNA was significantly reduced when METTL3 was downregulated (Figure 4(e)). Moreover, we also found that global m^6^A modification levels were reduced, COL10A1 protein expression and mRNA stability were all suppressed in CAFs upon METTL3 knockdown (Figures 4(d)–4(g)). Taken together, we speculate that the increased expression of COL10A1 in CAFs may be due to METTL3 stabilizing its expression by increasing m^6^A modification of COL10A1 mRNA.

### 3.5. METTL3 in CAFs Facilitates Malignant Behavior of SW900 by Mediating m^6^A Methylation of COL10A1

**Figure 5:**
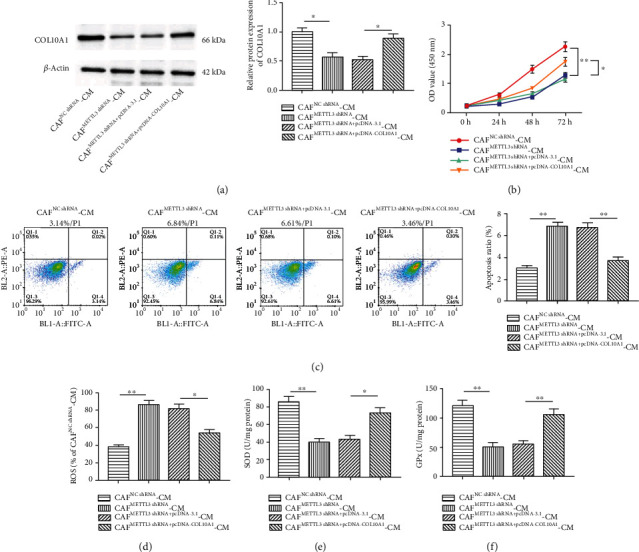
METTL3 in CAFs facilitates malignant behavior of SW900 by mediating m^6^A methylation of COL10A1. (a) Western blotting was applied to test COL10A1 protein level in SW900 cells treated with CAF^METTL3shRNA^-CM or/and CAF^METTL3shRNA+pcDNA-COL10A1^-CM. (b) CCK-8 assay was employed to test SW900 cell proliferation treated with CAF^METTL3shRNA^-CM or/and CAF^METTL3shRNA+pcDNA-COL10A1^-CM. (c) The annexin V-FITC/PI double staining assay was used to detect cell apoptosis in SW900 cells treated with CAF^METTL3shRNA^-CM or/and CAF^METTL3shRNA+pcDNA-COL10A1^-CM. (d–f) ELISA was employed to examine contents of ROS, SOD, and GPX in SW900 cells treated with CAF^METTL3shRNA^-CM or/and CAF^METTL3shRNA+pcDNA-COL10A1^-CM. Data were presented as mean ± SEM. *N* = 3. ^∗^*P* < 0.05, ^∗∗^*P* < 0.01.

Next, we will further analyze whether the effects of CAFs on LUSC cells are through the METTL3/COL10A1 axis. METTL3 shRNA was transfected into CAFs alone or together with pcDNA-COL10A1, and SW900 cells were then cultured with CAF-CM. The results demonstrated that COL10A1 expression was downregulated (Figure 5(a)), cell proliferation (Figure 5(b)) and SOD (Figure 5(e)), and GPX (Figure 5(f)) contents were decreased, and the rate of apoptosis (Figure 5(c)) and ROS production (Figure 5(d)) were increased in SW900 cells after knockdown of METTL3 in CAFs, these above results being partially counteracted by COL10A1 overexpression.

### 3.6. COL10A1 Interference Impairs CAF Acceleration of LUSC Xenograft Tumor Growth in Nude Mice

**Figure 6:**
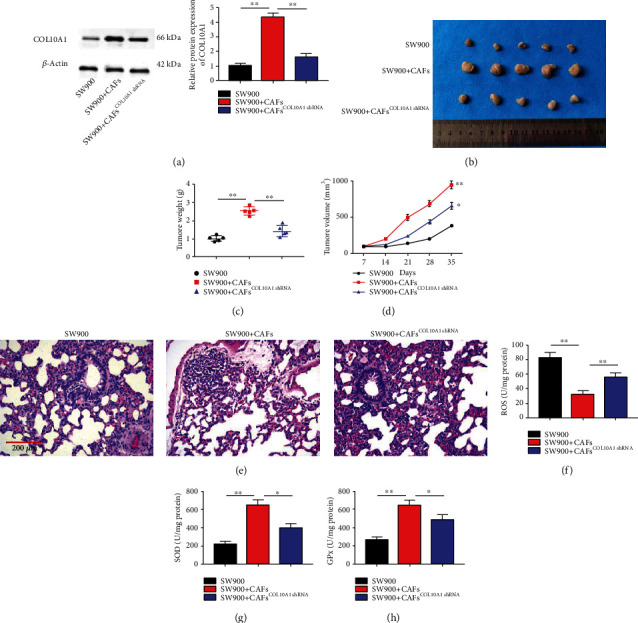
COL10A1 interference impairs CAF acceleration of LUSC xenograft tumor growth in nude mice. (a) Western blotting was applied to test COL10A1 protein level in mice from each group. (b) Representative images of tumors from each group; (c) Tumor growth curves; (d) Tumor mass of each group; (d) HE staining was applied to detect lung injury in mice from each group. (f–h) ELISA was used to test the production of ROS, SOD, and GPX in mice serum. Data were presented as mean ± SEM. *N* = 3. ^∗^*P* < 0.05, ^∗∗^*P* < 0.01.

Based on the *in vitro* findings, we confirmed that COL10A1 played a crucial role in CAFs promoting LUSC cell growth. Nude mice were subcutaneously injected with SW900 cells or/and CAFs stably transfected with a COL10A1 shRNA plasmid vector to further evaluate the effect of CAF^COL10A1^ on LUSC cell growth *in vivo* (Figure 6(a)). SW900 and CAFs injection significantly increased tumor volume and mass compared with SW900 transplanted tumors, whereas tumor growth was inhibited after CAF^COL10A1shRNA^ injection, with a size close to that of the untreated group (Figures 6(b)–6(d)). Besides, the results of HE staining of mouse lung tissues suggested that large clumps were found in the SW900+CAFs group, whereas the clumps became smaller in the SW900+CAFs^COL10A1shRNA^ group (Figure 6(e)). Consistent with the *in vitro* results, ROS production was decreased and contents of SOD and GPX were increased in the SW900+CAFs group compared with the SW900 group, whereas the above results were significantly reversed after knockdown of COL10A1 in CAFs (Figures 6(f)–6(h)).

## 4. Discussion

LUSC is one of the most common malignant tumors of the lung tissues. Despite continuous improvements in medical technology in recent years, the 5-year survival rate of LUSC remains less than half [22]. Therefore, it is still an important task to study its specific mechanism. In recent years, researchers have gradually found that TME as an ‘inner person' rather than an ‘outer person' is involved in most stages of tumor progression during the development of tumors [23]. Among them, CAFs, the most predominant stromal cells in the TME, can directly or indirectly affect surrounding parenchymal tumors. Currently, CAFs are commonly identified by the combined detection of several of the following proteins, including *α*-SMA, asporin, FAP, PDGFR, and FSP1 [24]. In this study, we compared extracted primary CAFs and NFs and found that expression of FAP and *α*-SMA were substantially increased in CAFs. CAFs have been shown to enhance squamous cell carcinoma (SCC) growth, but it is unclear whether they promote SCC lung metastasis. One data showed that TRAP1, an important regulatory molecule for glucose metabolism in CAFs, was able to promote oral squamous cell carcinoma progression by regulating OXPHOS in CAFs [25]. LAMC1 promoted CXCL1 secretion, which stimulated inflammatory CAF formation via CXCR2-pSTAT3, which in turn accelerates esophageal squamous cell carcinoma progression [26]. Ba et al. demonstrated that CAF-CM facilitated proliferation of tongue squamous cell carcinoma cells, and xenograft models confirmed this effect [27]. Subsequently, co-culturing the cells with NF/CAF-CM revealed that the proliferative capacity of the SW900 and LOU-NH91 cells was significantly enhanced, and the apoptotic rate and oxidative stress levels were reduced after treatment with CAF-CM, indicating that CAFs can encourage the proliferation of LUSC.

COL10A1 belongs to the *α*-chain of the collagen family collagen X. In mammals, collagen is one of the most abundantly expressed proteins [28]. It has been reported in the literature that the collagen superfamily consists of 28 types of collagens, including collagen I to collagen XXVIII [29]. COL10A1 plays a considerable role in endochondral ossification, possibly related to matrix degradation, calcification, vascular invasion, and mutations in this gene are associated with metaphyseal chondrodysplasia [30]. It was demonstrated that COL10A1 expression preceded the development of bone vascularization and then altered the properties of the extracellular matrix contributed to the invasion of blood vessels, and angiogenesis was particularly important for tumor tissues [31]. Thus, COL10A1 is a specific marker potentially involved in tumor angiogenesis [32]. It has been reported that COL10A1 is highly expressed in most tumor tissues, such as breast cancer [33], colorectal cancer [34], and gastric cancer [35] and so on. Up-regulation of COL10A1, a molecular marker of early colorectal cancer, encouraged colorectal cancer cell proliferation, migration, and invasion [36]. Logistic regression analysis indicated that high expression of COL10A1 in gastric cancer was largely associated with pathological stage, tumor differentiation, and T classification [37]. Bidirectional communication between tumor cells and CAFs regulates extracellular matrix (ECM) deposition and remodeling. As a result of this dynamic process, soluble ECM proteins can be released into the bloodstream and may represent novel circulating biomarkers for cancer diagnosis. Circulating COL11A1 expression is elevated in plasma samples of breast cancer patients and may be helpful in the diagnostic evaluation of suspicious breast nodules [38]. Moreover, a research confirmed that the expression of circulating protein COL10A1 was substantially elevated in the plasma of lung cancer patients compared with lung cancer cells [39]. In the present research, with the help of GEO dataset analysis and screening possible key genes, COL10A1 was found to be significantly higher expressed in LUSC patient plasma-derived CAFs compared with NF and LUSC cells. Furthermore, knockdown of COL11A1 was able to counteract the accelerating effect of CAF-CM on the malignant behavior of the cells.

Epigenetics refers to the study of heritable and reversible phenotypic changes that do not involve alterations in the nuclear DNA sequence [40]. It mainly includes DNA methylation, RNA modification, noncoding RNAs, and histone modification. RNA methylation includes N1 methyladenine (m^1^A), 5-methylcytosine (m^5^C), N6 methyladenine (m^6^A), and N7 methylguanine (m^7^G) [41]. Among these, m^6^A is the most common and most abundant RNA modifier in eukaryotic cells and can occur in messenger RNA (mRNA), ribosome RNA (rRNA), transfer RNA (tRNA), and noncoding RNA (ncRNA) [42]. M^6^A refers to the methyl modification that occurs at the sixth position of adenine (a) under the action of methyltransferases, and is the most abundant modification occurring post transcriptionally in mRNA, accounting for about 50%, mainly occurring near stop codons and 3 ‘untranslated regions' [43]. A research demonstrated that chronic Cr exposure could alter cellular epi transcriptome by adding m^6^A RNA modification through elevating the RNA methyltransferase METTL3 expression, which plays an important role in chronic Cr exposure-induced cell transformation, cancer stem cell-like property, and lung tumorigenesis [44]. METTL3 acts as an oncogene in NSCLC by mediating Bcl-2 mRNA m^6^A modification, suggesting that targeting METTL3 may be an effective therapeutic strategy for the clinical management of NSCLC [45]. Further, Pan et al. demonstrated that CAFs derived exosomes suppress 5-Fluorouracil sensitivity in colorectal cancer cells via the METTL3/miR-181d-5p axis [46]. Combined with the above findings, this experiment confirmed that the expression of METTL3 increased in LUSC derived CAFs. Besides, we speculate that the increased expression of COL10A1 in CAFs may be due to METTL3 stabilizing its expression by increasing m^6^A modification of COL10A1 mRNA. METTL3 in CAFs promotes proliferation and inhibits oxidative stress-induced apoptosis in LUSC cells by mediating m^6^A methylation of COL10A1.

Taken together, the results of this study indicate that LUSC-derived CAFs have the ability to accelerate cell proliferation and repress oxidative stress-induced apoptosis *in vitro* and *in vivo*. The main mechanism is upregulation of m^6^A modification of COL10A1 by an increase in METTL3 expression, which stabilizes COL10A1 expression for subsequent delivery into LUSC cells. These findings contribute to a better understanding of the molecular biological mechanisms by which CAFs promote LUSC and hopefully provide new molecular targets and theoretical basis for LUSC therapy. Nevertheless, this research content is mainly based on the conditioned medium of CAFs and lacks further confirmation on exosomes, which will be the focus of our subsequent studies.

## Data Availability

The labeled dataset used to support the findings of this study is available from the corresponding author upon request.
